# Supplementary material for: Impact of C‐terminal amino acid composition on protein expression in bacteria
Source: Mol Syst Biol. 2020 May 25;16(5):e9208. doi: 10.15252/msb.20199208 (PMC7246954; doi:10.15252/msb.20199208)
Supplement: Supplementary file 1 — Appendix [file MSB-16-e9208-s001.pdf]

Appendix

Table of Contents

Figure S1..2
Figure S2..3
Figure S3..4
Figure S4..5
Figure S5..6
Figure S6..7
Figure S7..8
Figure S8..9
Figure S9..10
Figure S10..11
Figure S11..12
Figure S12..13
Figure S13..14
Figure S14..15
Figure S15..16
Figure S16..17
Figure S17..17
Figure S18..18
Figure S19..19
Figure S20..20
Figure S21..21

Figure S1

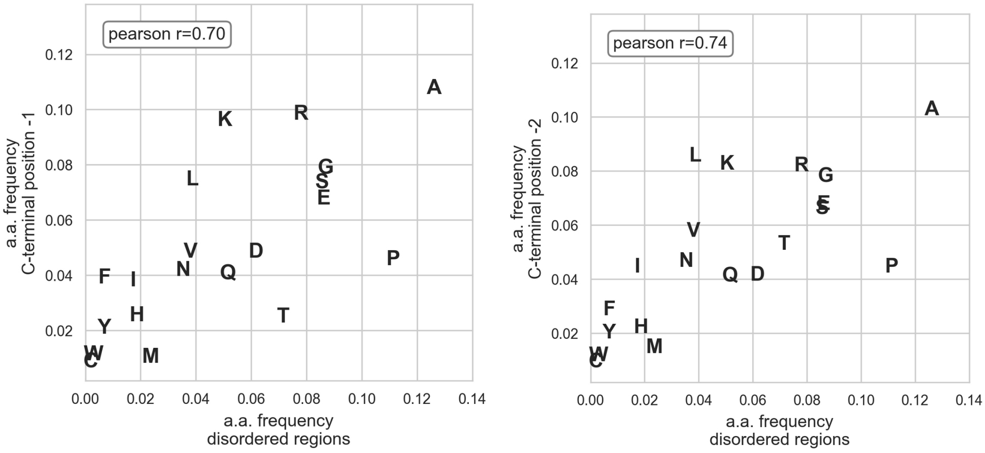

Figure S1. Correlation between amino acid frequency at C-terminal and in disordered regions. The amino acid frequencies were computed for all disordered regions in bacteria.

Figure S2

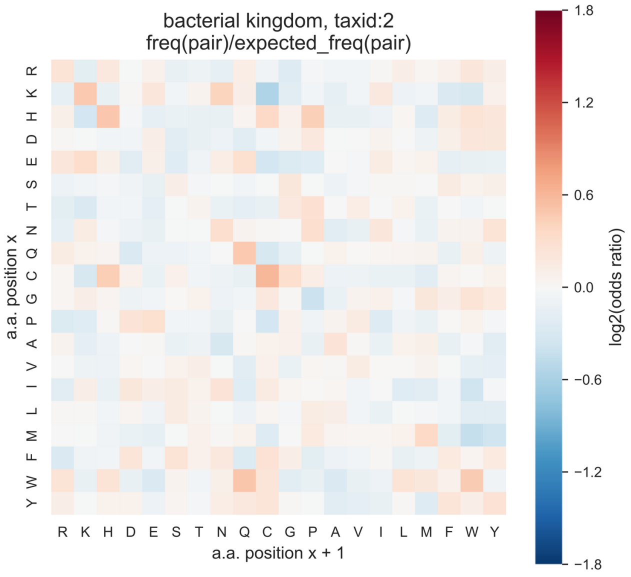

Figure S2. Biases (epistasis) in frequency of amino acid pairs in the bulk in the bacterial kingdom. We computed the odds ratio of observed amino acid pairs in the bulk compared to their expected frequency if they were independent (see Methods).

Figure S3

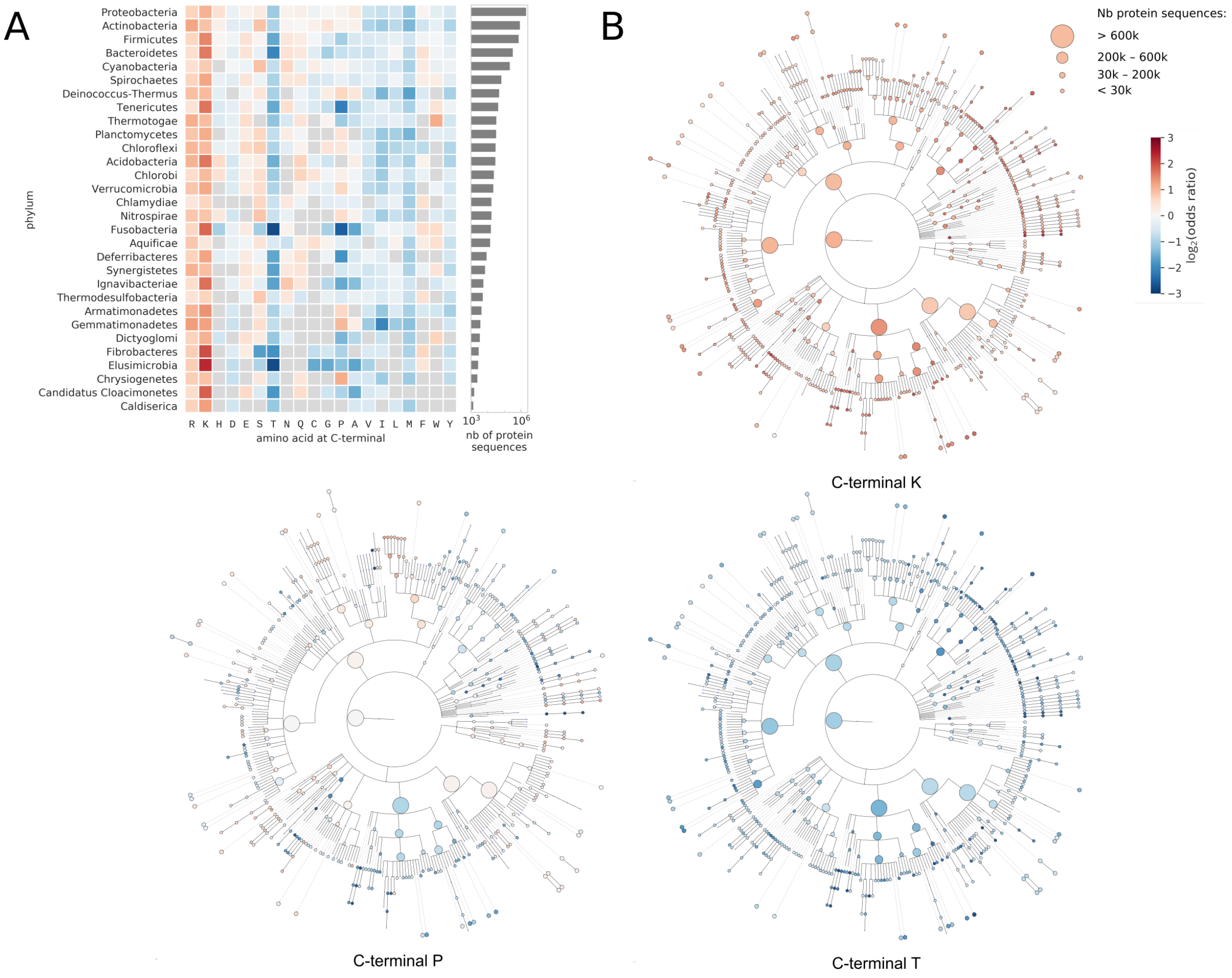

Figure S3. Biases in C-terminal amino acid composition across the bacterial taxonomic tree. (A) Distribution of amino acid bias for all phyla. (B) Distribution of the amino acid bias of lysine, proline and threonine at C-terminal across the taxonomic tree. The tree was cut down to the family rank (674 nodes out of 4083), except for species which do not belong to any family. The enrichment for lysine and depletion of threonine are present in the vast majority of the taxonomic clades, while the biases for proline varies greatly, showing positive and negative biases depending on the clade.

Figure S4

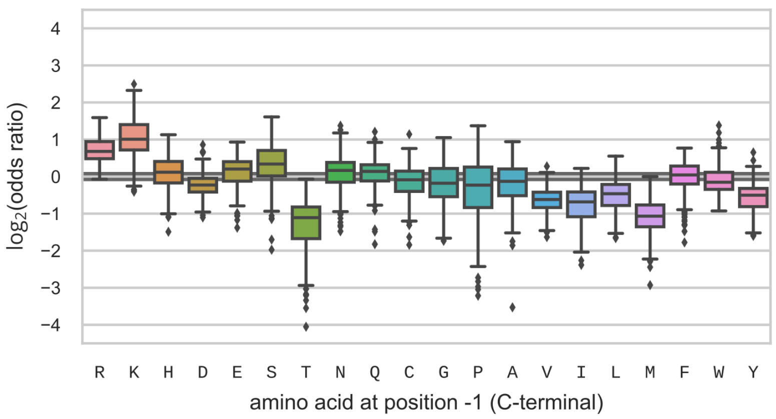

Figure S4. Biases in C-terminal amino acid composition for all families. The biases in amino acid frequencies at position -1 were computed against the frequency in the bulk, for each of the 280 taxonomic clades at the family rank. Bias is reported as the log2(odds ratio), without controlling for statistical significance. Some amino acids, as Lys and Thr, exhibit a systematic bias in the vast majority of the taxonomic clades.

Figure S5

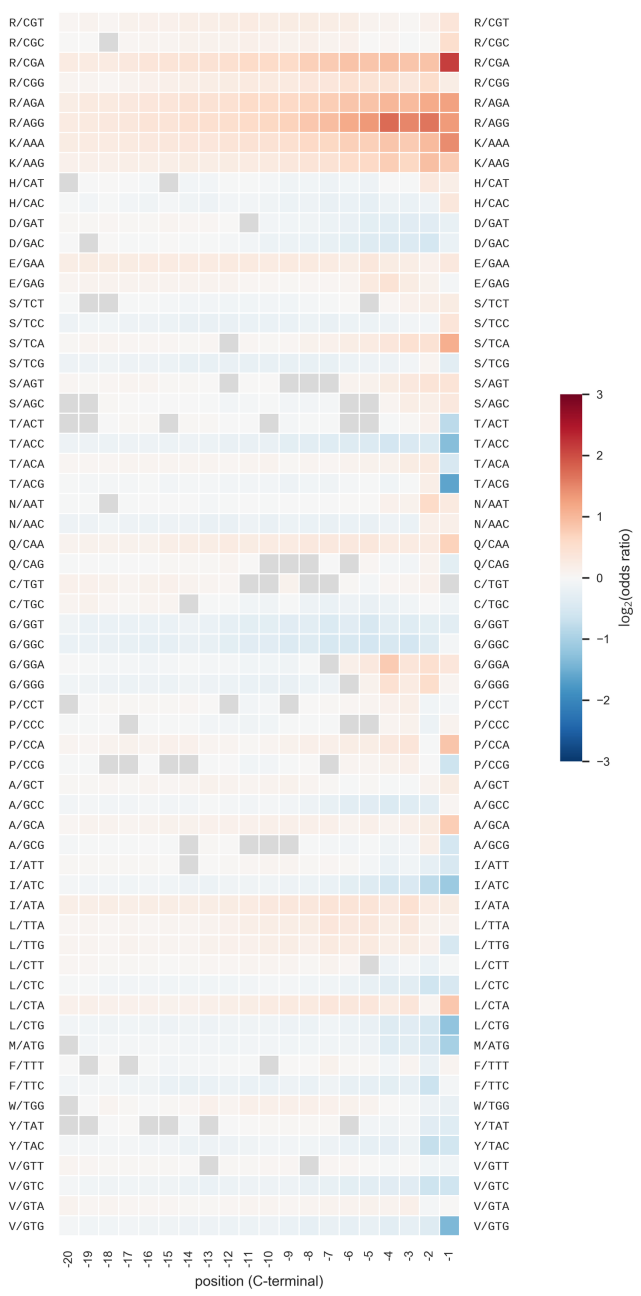

Figure S5. Position-specific biases in C-terminal codon composition in the bacterial kingdom. Codon composition at the C-terminal of bacterial protein sequences shows higher (red) or lower (blue) frequency when compared to their frequency in the bulk of the sequence (same color code for all panels). Significance of the biases are tested using exact Fisher test and multiple-tests correction with 5% false discovery rate.

Figure S6

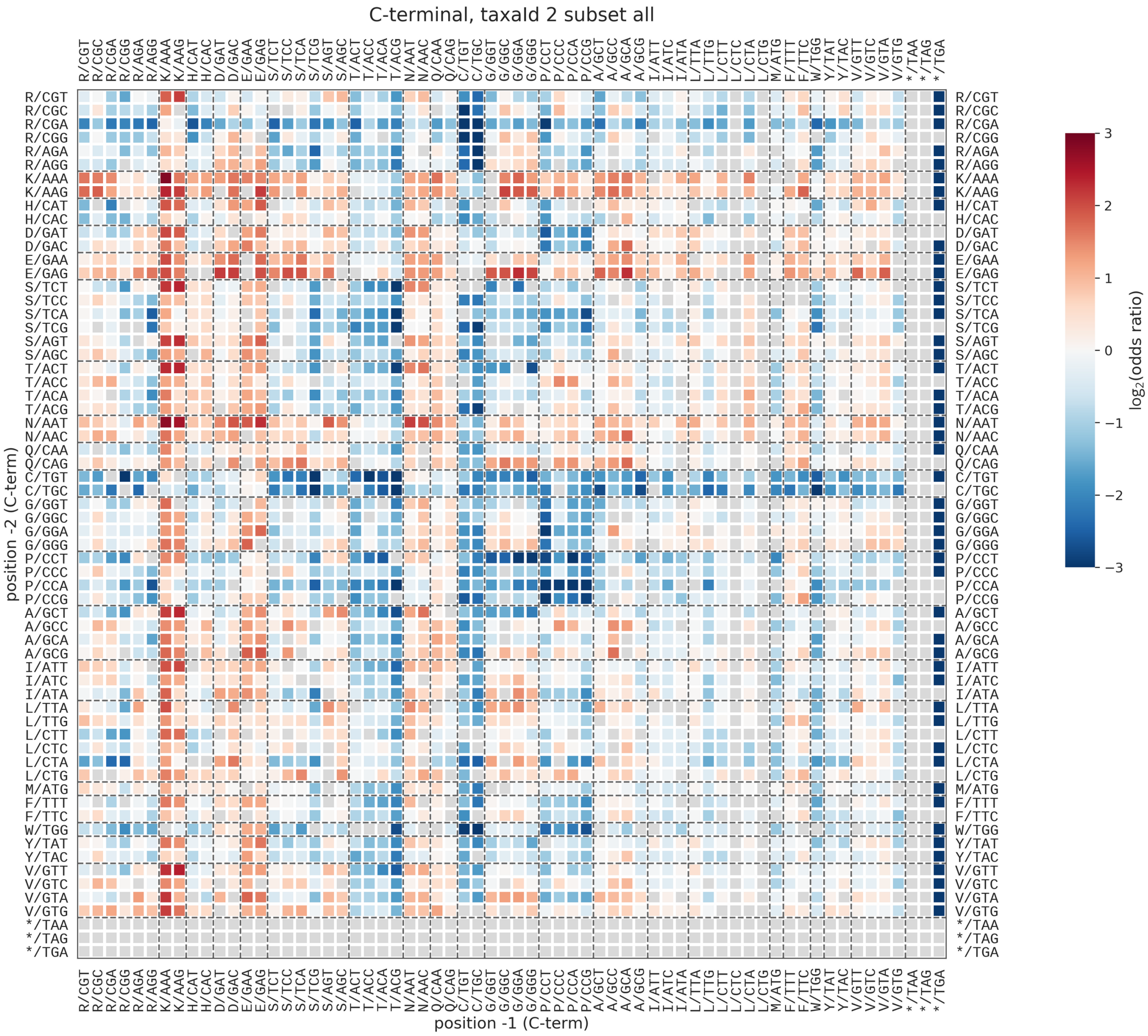

Figure S6. Biases in C-terminal protein sequence composition in the bacterial kingdom at the level of codon pairs. The composition of the last two codons at the C-terminal of bacterial protein sequences shows higher (red) or lower (blue) frequency when compared to the frequency of codon pairs in the bulk of the sequence. Significance of the biases are tested using exact Fisher test and multiple-tests correction with 5% false discovery rate.

Figure S7

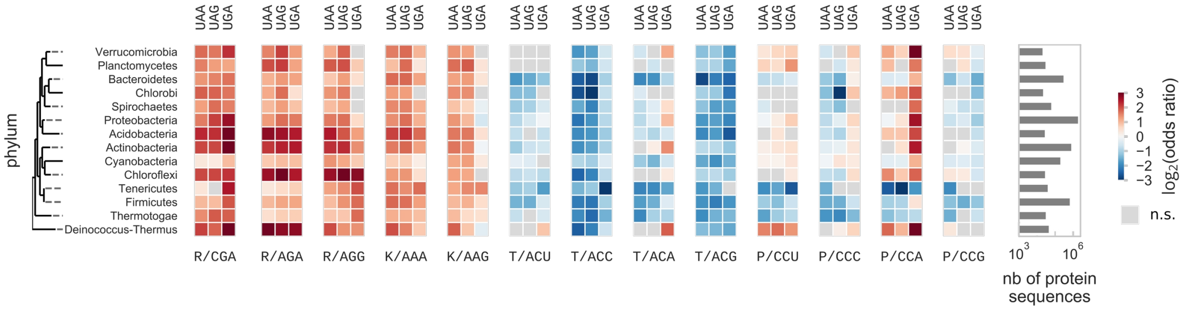

Figure S7. Codon composition biases at C-terminal and stop codon context. In the bacterial kingdom, protein sequences are first classified by their stop codon context. Codon frequency at C-terminal is then compared to the bulk codon frequency within each stop codon context class. Significance of the biases were tested using exact Fisher test and multiple-tests correction with 5% false discovery rate within each class. C-terminal codon biases in each stop codon context at the level of phyla, for a selection of codons that showed strong enrichment or depletion. Phyla were ordered following an approximate phylogenetic tree.

Figure S8

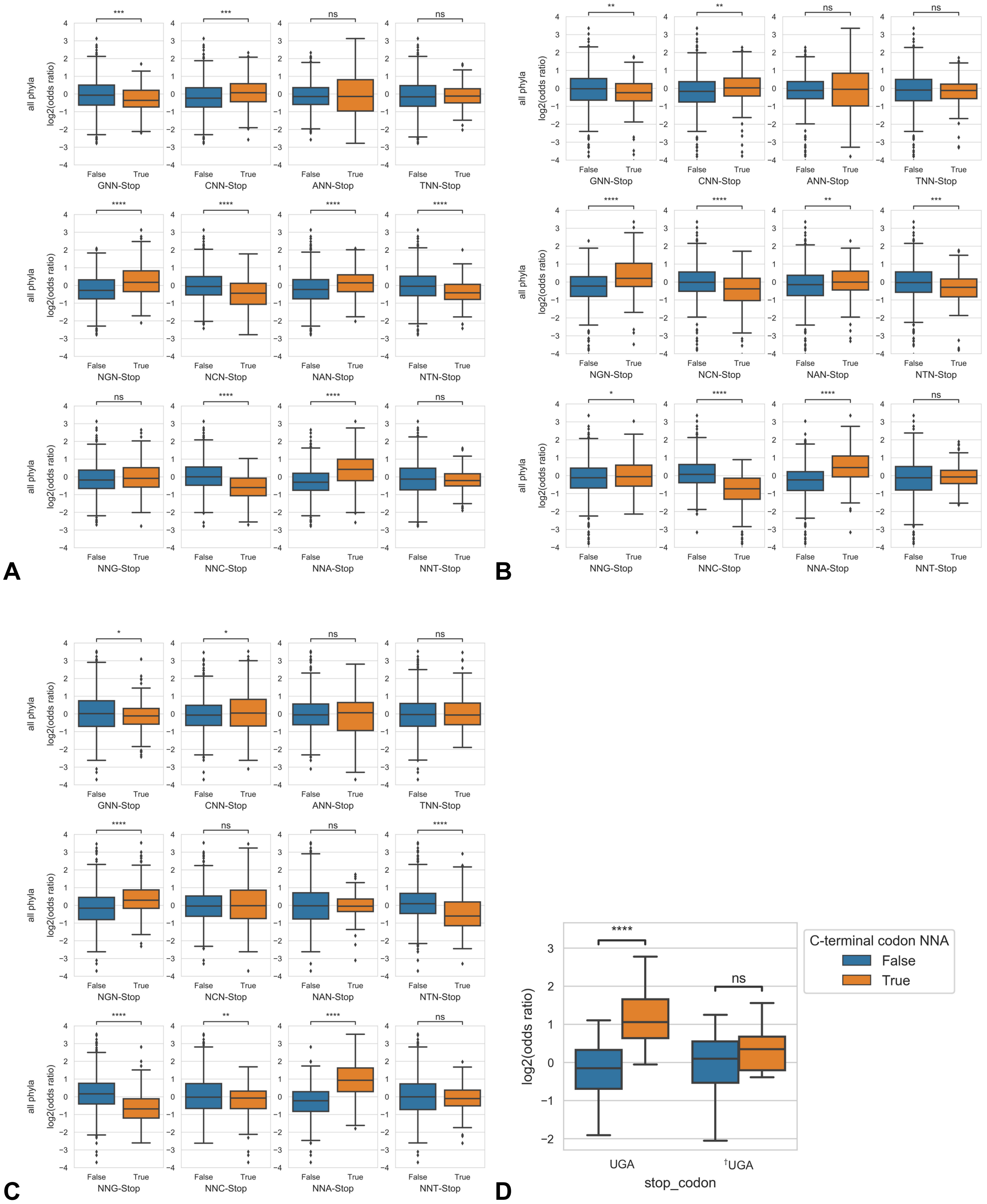

Figure S8. C-terminal codon bias in the three stop codon contexts for different nucleotide motifs. Distribution of the codon bias (log2 of odds ratio) at position -1 in the (A) UAA, (B) UAG, or (C) UGA stop codon context for the 14 phyla with at least 20k sequences. Codons were classified depending on the identity of the nucleotide at either of the three

positions. (D) Same analysis in the bacterial kingdom, with codons classified into NNA and other codons, in the UGA stop codon context, and in the †UGA context. The latter was defined as follows: in the case of the UGA stop codon context, we further excluded genes for which the start codon of the downstream gene was overlapping with the stop codon at nucleotide position -1, e.g. NNA-UGA where AUG is the downstream start codon. Comparison between NNA codons and others, independent t-test p=2.2e-08 for UGA context, p=0.12 for †UGA context. The distributions were compared using independent t-test. Significance code: n.s. not significant for p > 0.01, * for p < 0.05, ** for p < 0.01, *** for p < 0.001, **** for p < 1e-4.

Figure S9

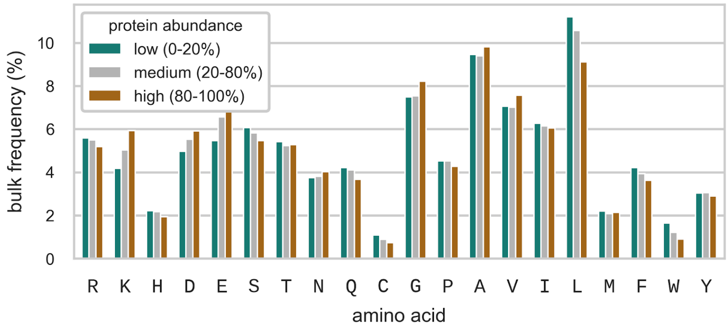

Figure S9. Amino acid bulk frequencies of proteins classified by abundance. Proteins were categorized into low (percentiles 0-20, green), medium (percentiles 20-80, grey) and high (percentiles 80-100, brown) abundance, and bulk frequency of amino acids for each of the abundance categories was computed.

Figure S10

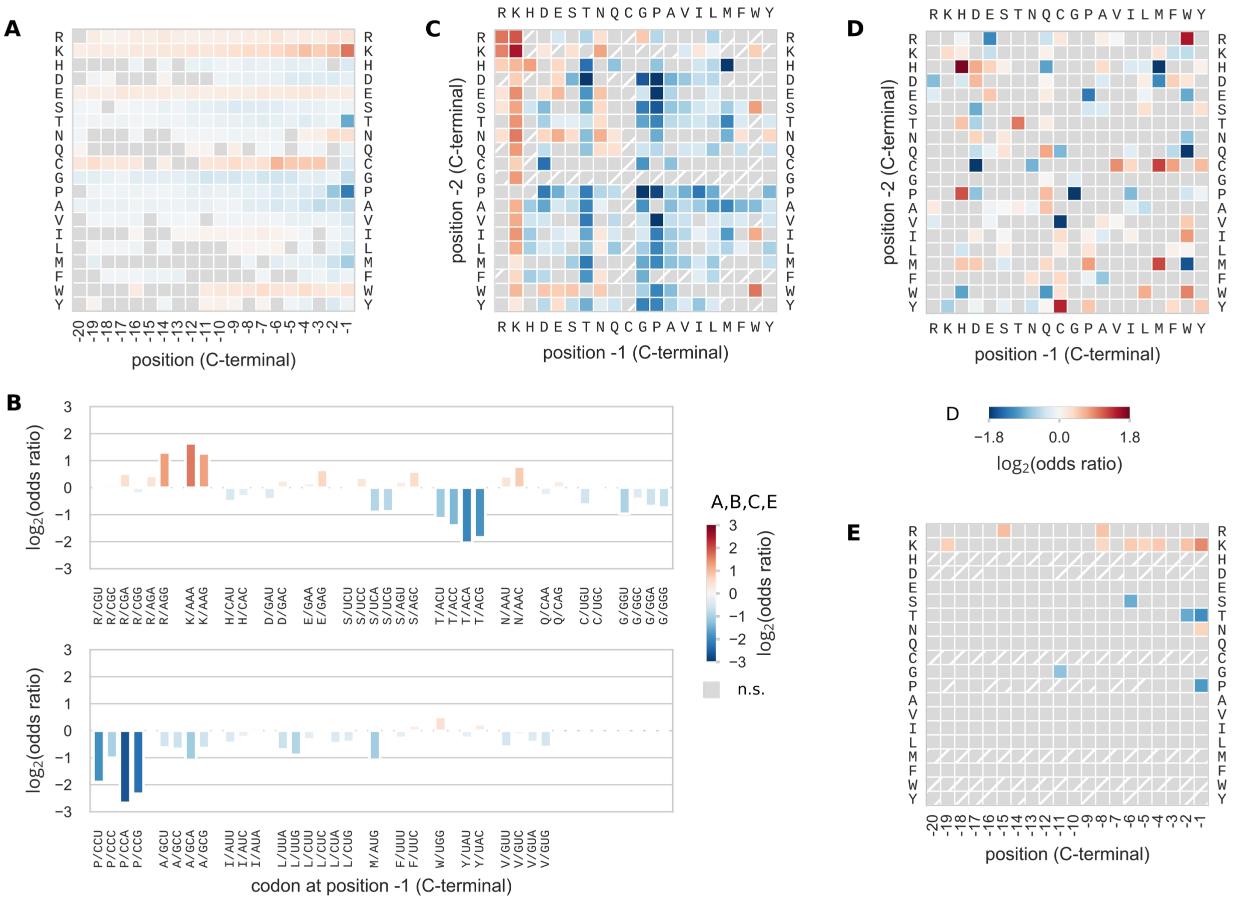

Figure S10. Biases in C-terminal amino acid and codon composition in the Mollicutes class and in the *Mycoplasma pneumoniae* species. Same analysis as in Fig. 1, for the Mollicutes class (A, B, C, D) and for the single species M. pneumoniae (E). Hashed pattern indicate a low number of cases which leads to low statistical power. The Mollicutes class contained 53 genomes and a total of 43250 protein sequences. In the case of the single species, low number of proteins sequences (660) prevented the detection of compositional biases for all amino acids.

Figure S11

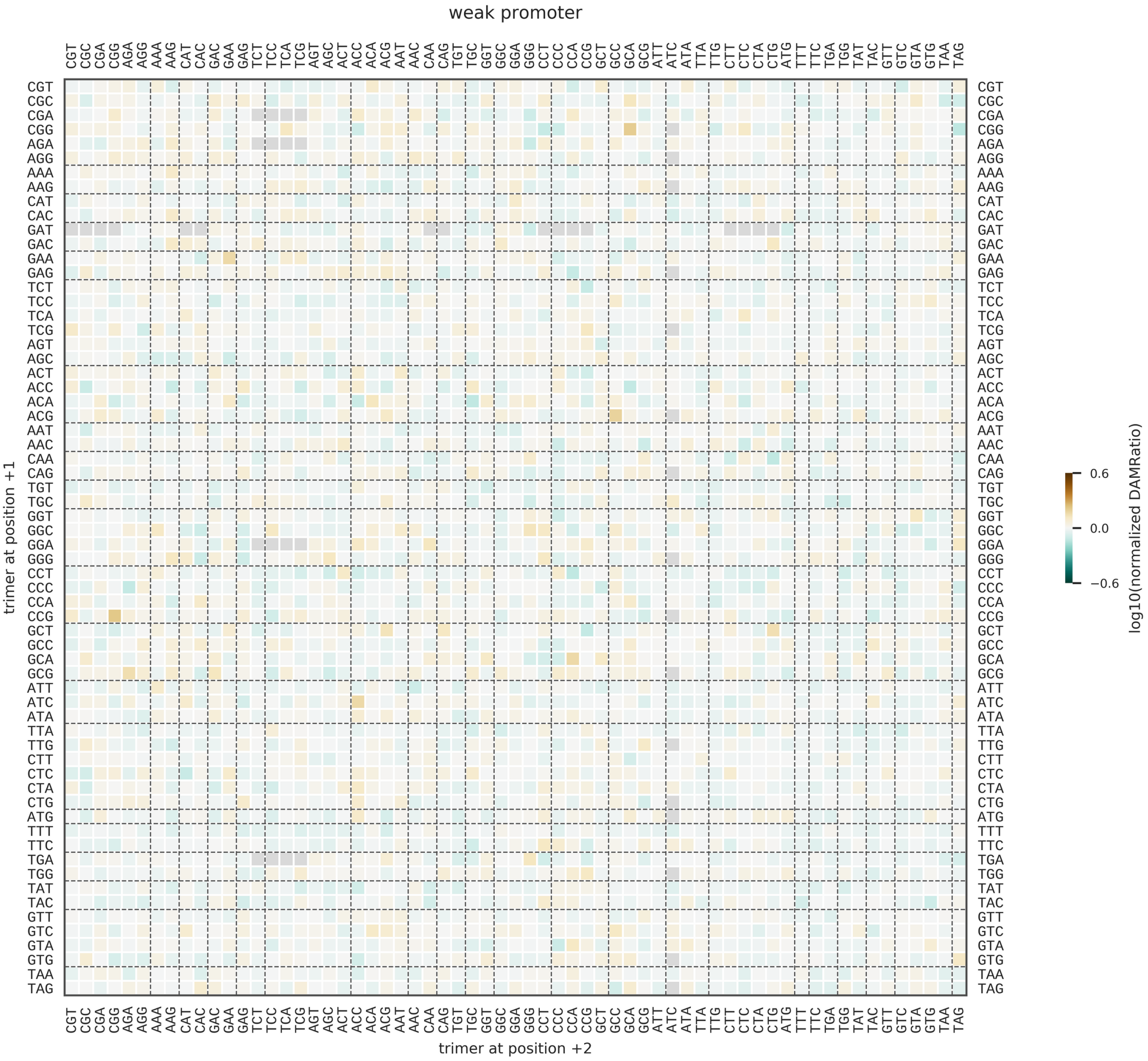

Figure S11. Effect of downstream hexamer on protein expression levels in the randomized C-terminal library with the weak promoter. Protein expression level readout is reported as the log10 of the DAM ratio relative to the average. The randomized hexamer is located just downstream of the stop codon, and should have little influence in the protein expression level. Hexamers that contained the GATC motif were filtered out (missing data, grey squares).

Figure S12

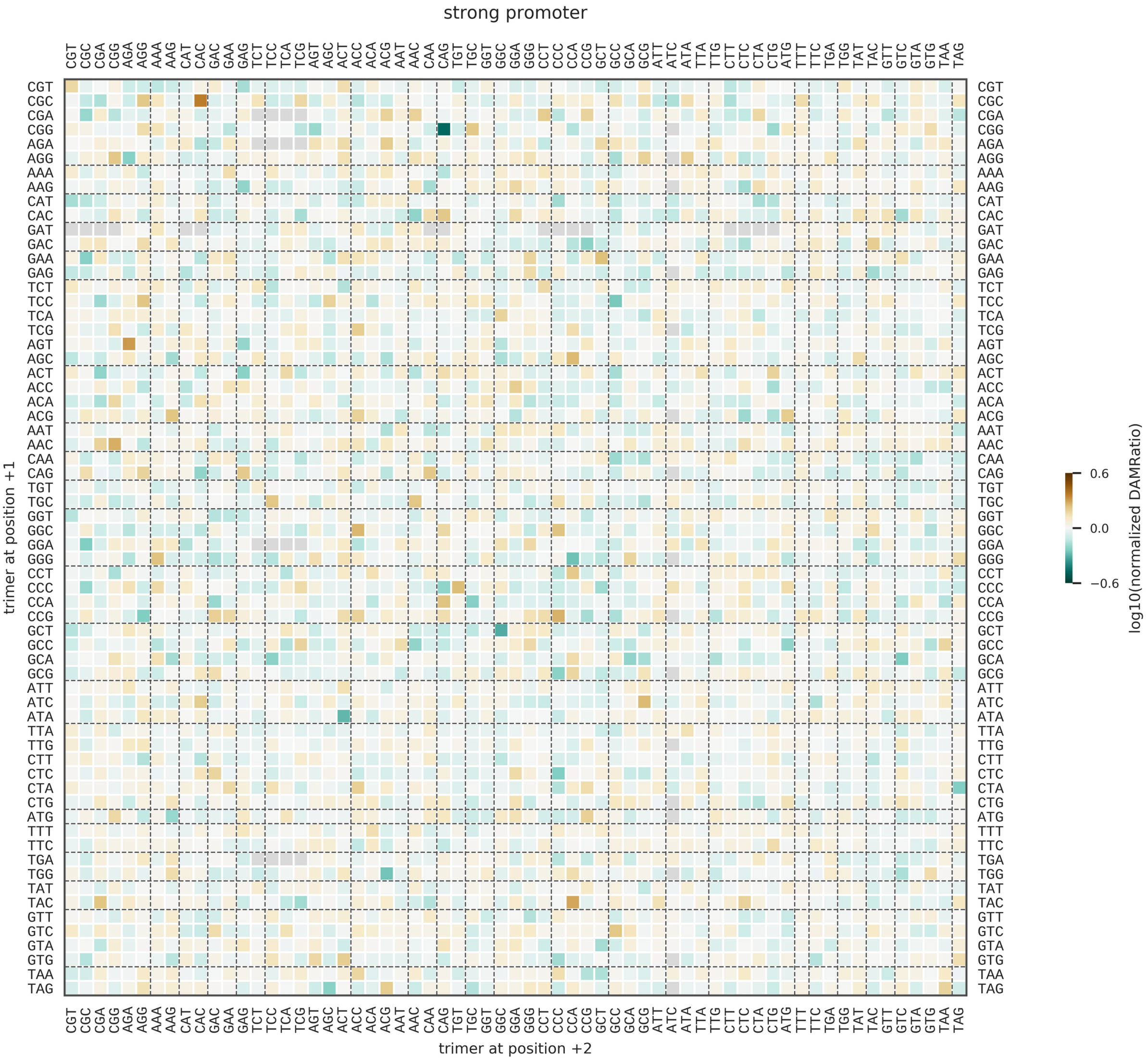

Figure S12. Effect of downstream hexamer on protein expression levels in the randomized C-terminal library with the strong promoter. Protein expression level readout is reported as the log10 of the DAM ratio relative to the average. The randomized hexamer is located just downstream of the stop codon, and should have little influence in the protein expression level. Hexamers that contained the GATC motif were filtered out (missing data, grey squares).

Figure S13

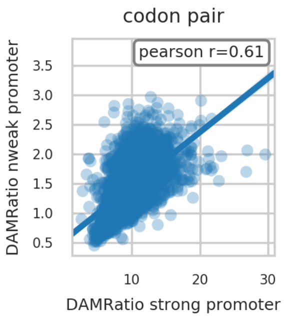

Figure S13. Correlation of DAMratio for the weak promoter library and the strong promoter library, at the level of C-terminal codon pair.

Figure S14

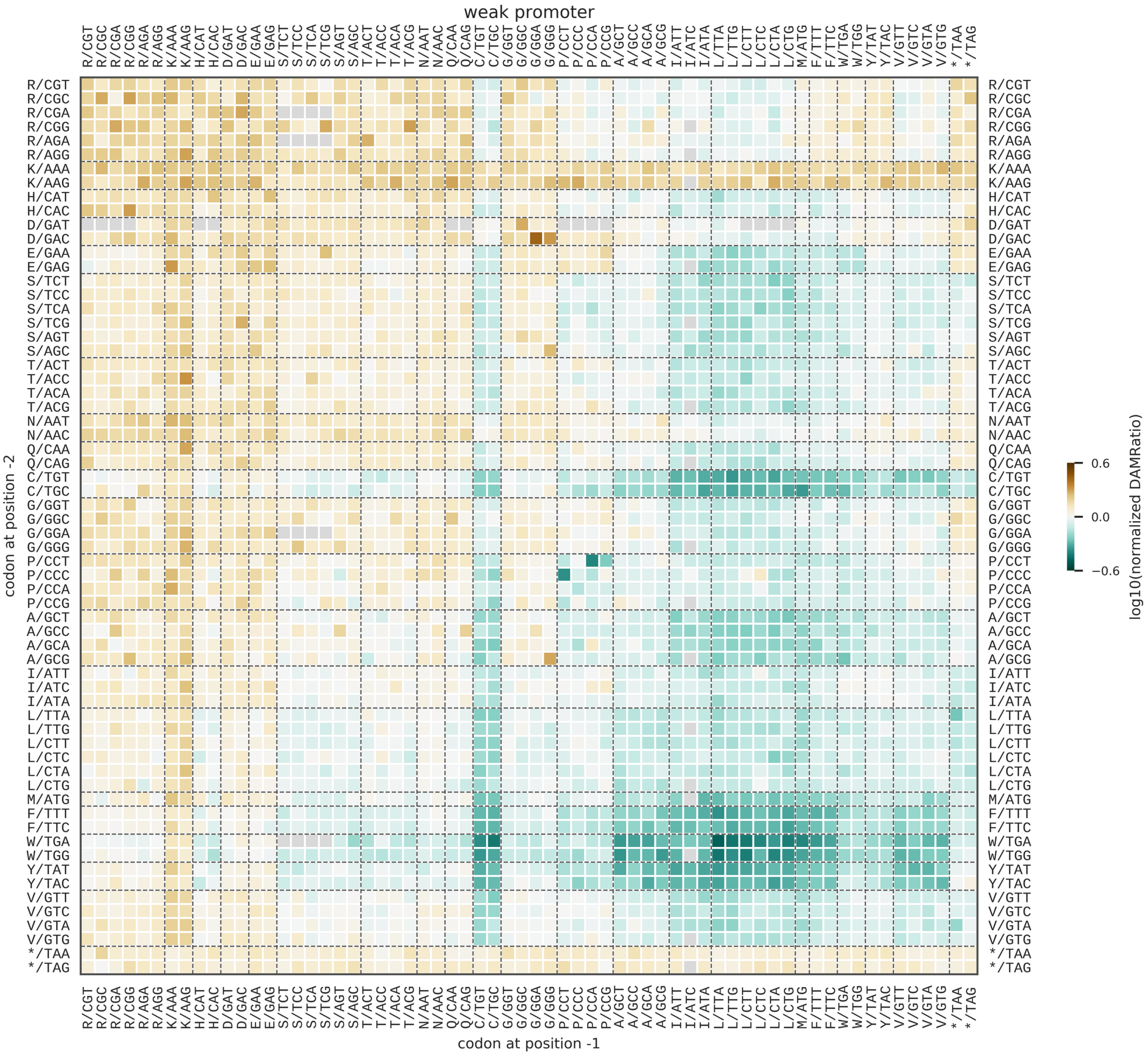

Figure S14. Effect of C-terminal codon pair on protein expression levels in the randomized C-terminal library with the weak promoter. Protein expression level readout is reported as the log10 of the DAM ratio relative to the average. Codon pairs that contained the GATC motif were filtered out (missing data, grey squares), for example CGA-TCT. The number of reads for sequences in each codon pair is much lower than in the analysis at the level of individual codons, which leads to a stronger influence of noise in the estimation of the DAMratio.

Figure S15

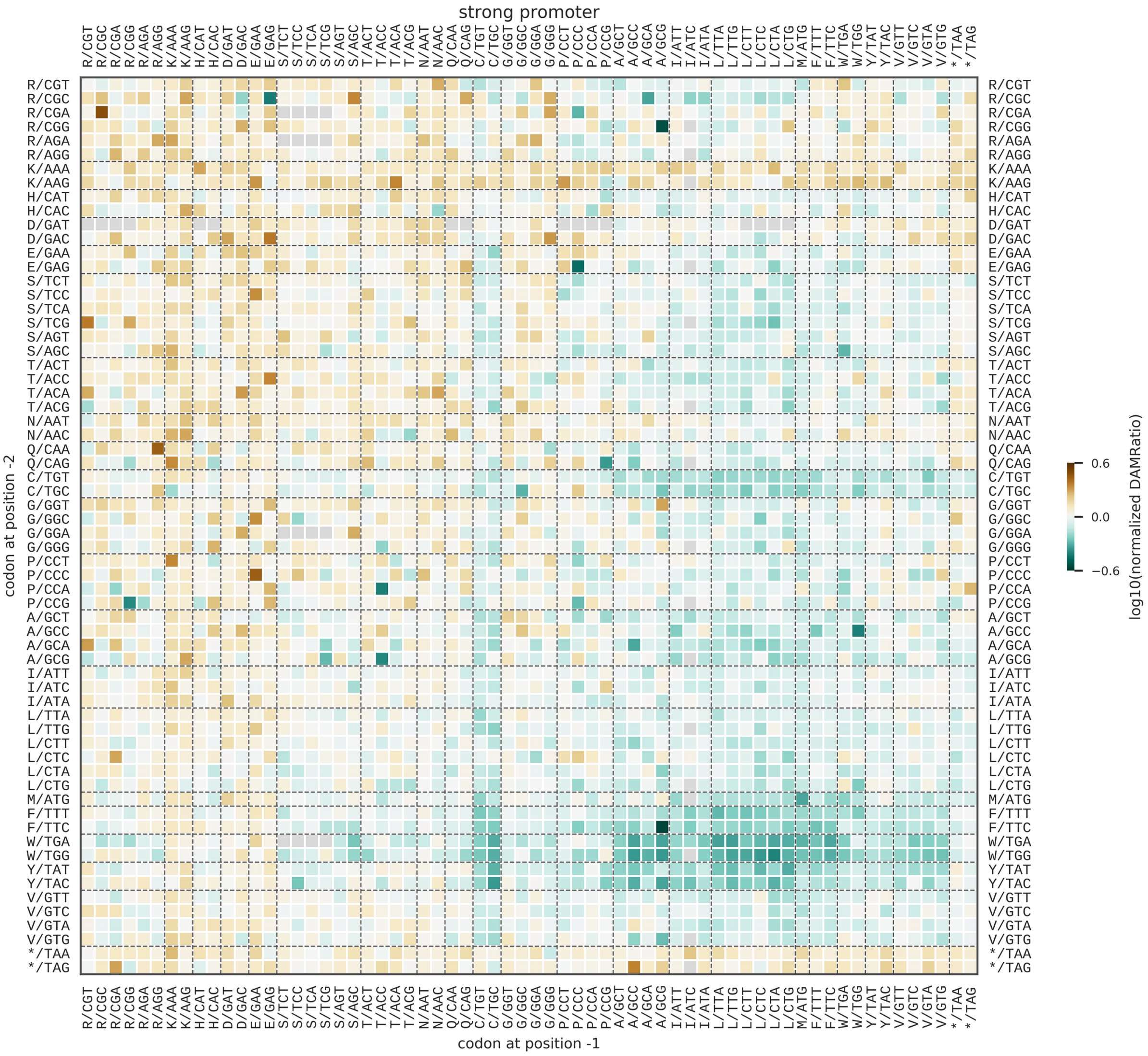

Figure S15. Effect of C-terminal codon pair on protein expression levels in the randomized C-terminal library with the strong promoter. Protein expression level readout is reported as the log10 of the DAM ratio relative to the average. Codon pairs that contained the GATC motif were filtered out (missing data, grey squares), for example CGA-TCT. The number of reads for sequences in each codon pair is much lower than in the analysis at the level of individual codons, which leads to a stronger influence of noise in the estimation of the DAMratio.

Figure S16

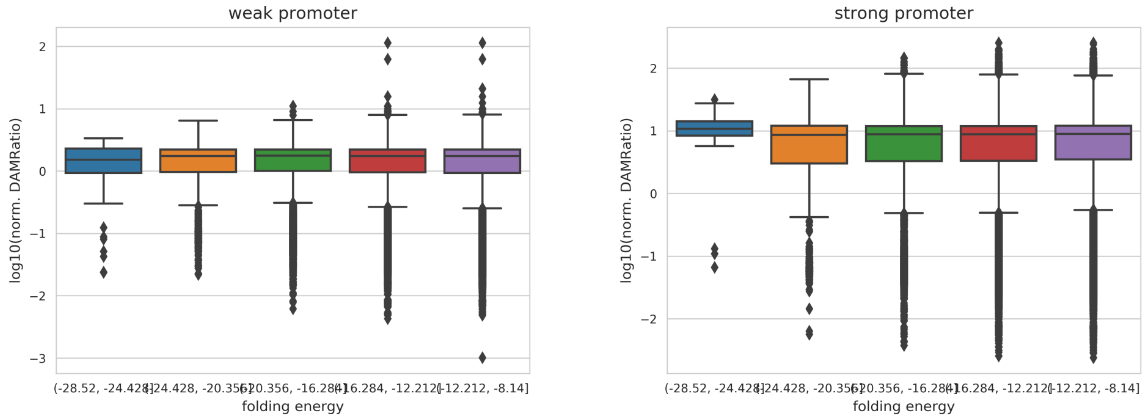

Figure S16. Distribution of the log10(DAMRatio) expression level as a function of the predicted folding energy of the C-terminal region for the randomized library. Folding energy was computed in a window ranging from 30 nucleotides upstream to 37 nucleotides downstream the randomized C-terminal sequence, both for the weak (panel A) and the strong (panel B) promoter. Sequence variants were then classified into 4 equal bins of folding energy values and their expression level distribution plotted. In general, folding energy variation was very small upon hexamer sequence variation, and expression levels did not show any dependence on folding energy.

Figure S17

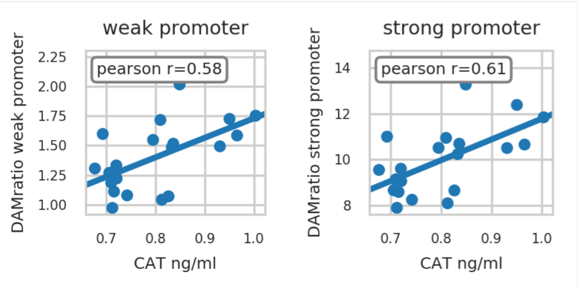

Figure S17. Impact of C-terminal codon on protein expression, correlation between expression levels in Cat reporter and ELM-seq experiments. The expression levels as measured in the cat reporter experiment are compared to the average DAMRatio of the corresponding C-terminal codon in the ELM-seq experiment, for the weak and strong promoters.

Figure S18

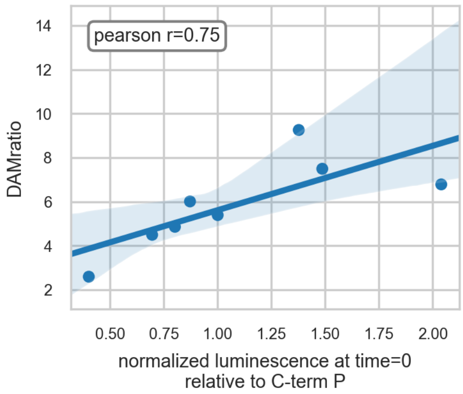

Figure S18. Correlation between C-terminal variants protein expression levels between the ELM-seq assay and the luciferase assay. The average expression levels as measured in the ELM-seq experiment (DAMratio), averaged for both weak and strong promoter libraries, are compared to the normalized luciferase luminescence as measured in the luciferase assay, averaged over the three replicates.

Figure S19

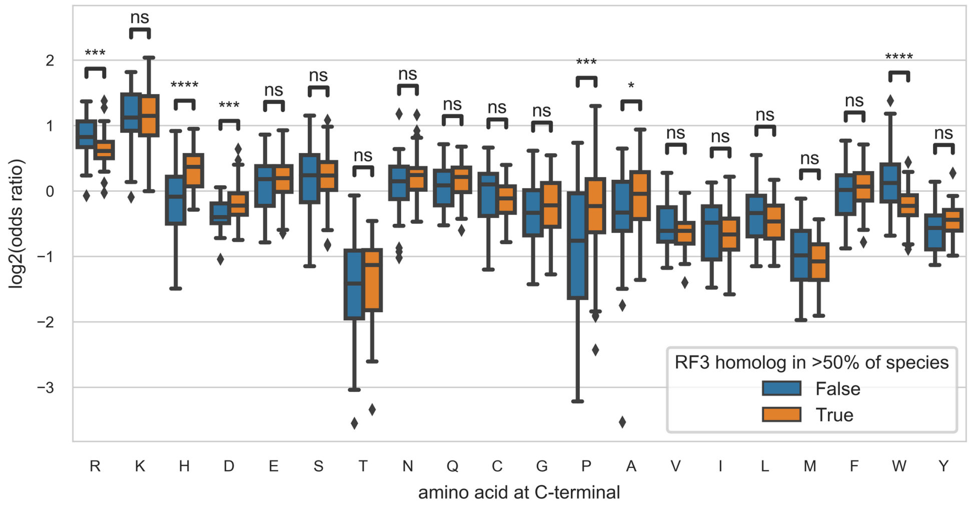

Figure S19. Changes in C-terminal amino acid biases for taxonomic clades classified by the presence of release factor 3 (RF3) homolog. We chose 114 clades at the family rank that contained at least 4 genomes in our database, and identified the species that contained the *prfC* homolog (RF3) based on EggNOG orthologous group assignment (OG id 05C8A). In total 990 proteins were annotated as *prfC* homolog in our database. Then, we classified each clade based on the proportion of species that contained the *prfC* homolog. We compared the distribution of C-terminal amino acid biases of clades with *prfC* presence in more than 50% of species, or less than 50% of the species. Differences in the mean of the biases for each amino acid was compared between the two classes using independent t-test.

Figure S20

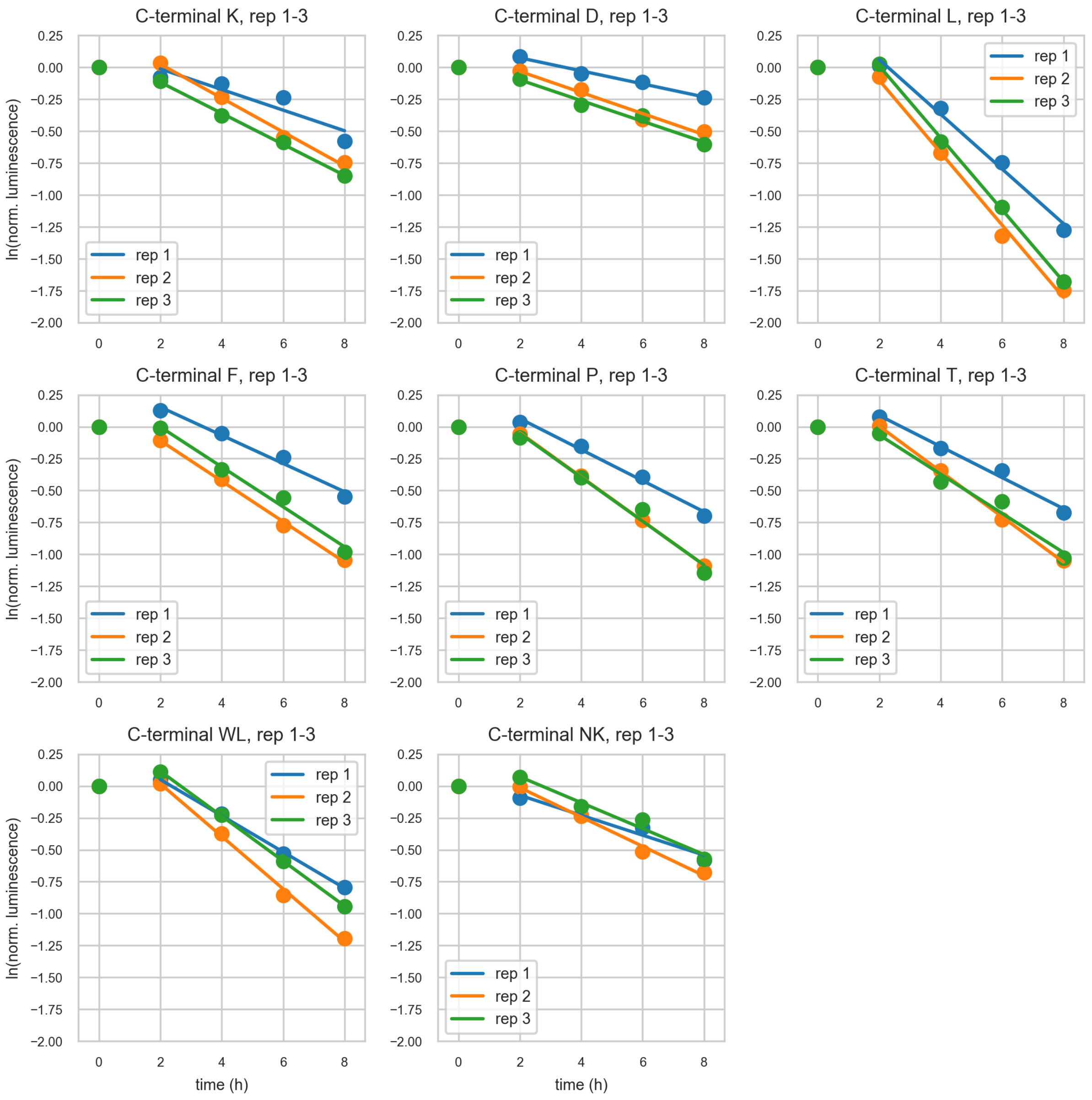

Figure S20. Protein degradation assay, fit to the exponential decay. For each C-terminal variant, the normalized luminescence at time points 2h, 4h, 6h and 8h was fitted to an exponential decay, independently for each of the three replicates.

Figure S21

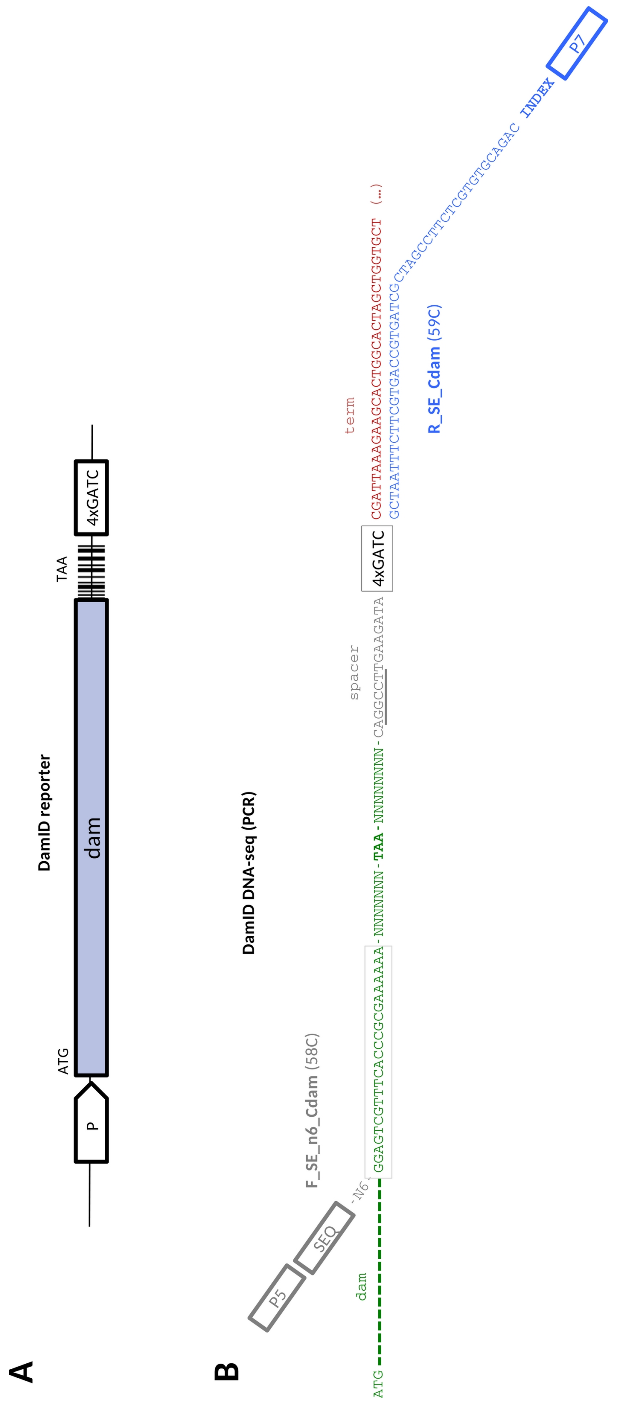

Figure S21. C-terminal screen ELM-seq damID sequencing scheme. A) Construct design: the C-terminal amino acids are randomized in frame. B) DamID: DNA-seq was performed by PCR amplification of the screen cassette (after digestion with GATC methylation sensitive or insensitive enzymes). The custom PCR oligos included the Illumina sequencing, flow-cell binding and index sequences.
