# Supplementary material for: Impact of C‐terminal amino acid composition on protein expression in bacteria
Source: Mol Syst Biol. 2020 May 25;16(5):e9208. doi: 10.15252/msb.20199208 (PMC7246954; doi:10.15252/msb.20199208)
Supplement: Supplementary file 2 — Expanded View Figures PDF [file MSB-16-e9208-s002.pdf]

Expanded View Figures

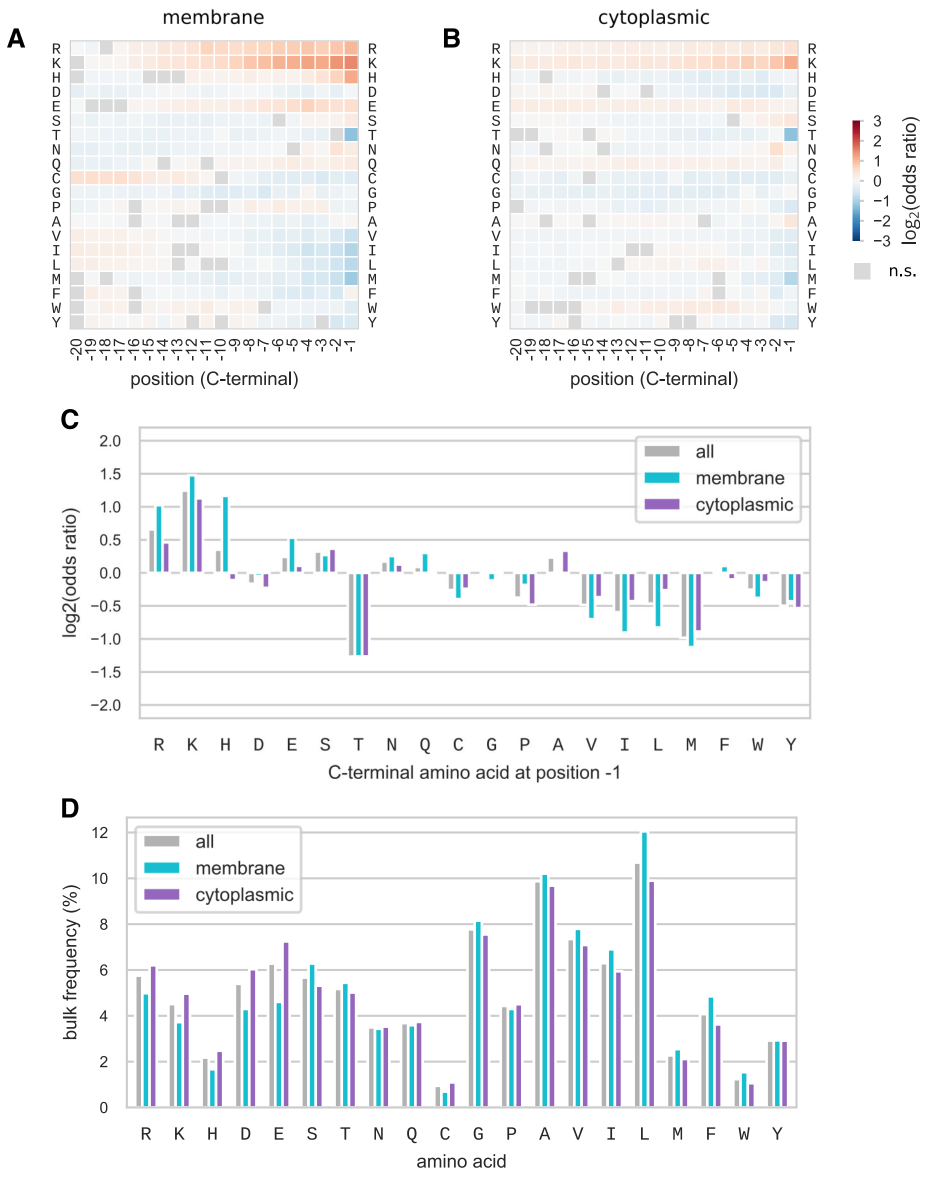

Figure EV1. C-terminal amino acid composition bias for membrane proteins.

A–D Proteins were classified as membrane or cytoplasmic proteins based on predicted subcellular localization, for a selection of 364 bacterial species. Position-specific C-terminal amino acid composition bias for membrane (A) and cytoplasmic (B) proteins. Significance of the biases was tested using Fisher's exact test and multiple testing correction with 5% false discovery rate. (C) Bias in amino acid composition at C-terminal (position −1) for membrane, cytoplasmic, and all proteins. (D) Amino acid bulk frequency.

© 2020 The Authors

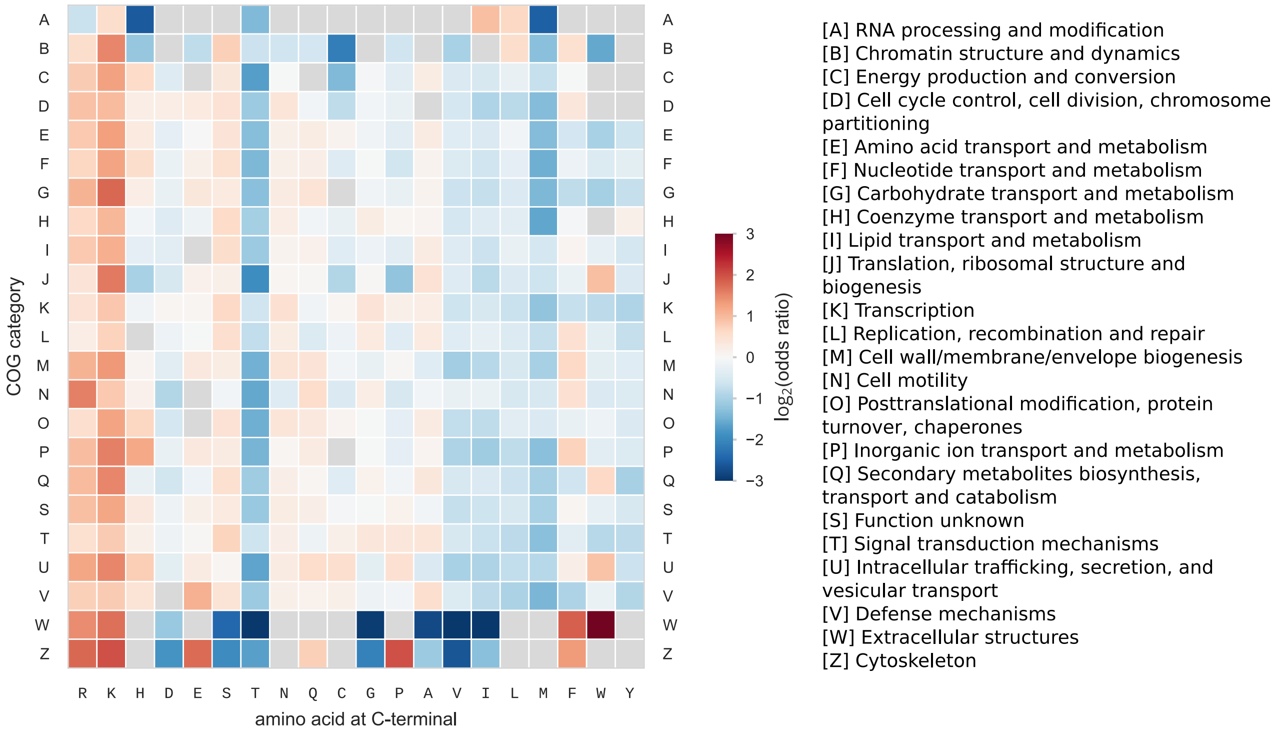

Figure EV2. C-terminal amino acid composition bias at position −1 for each COG category.

Proteins were classified based on their computationally assigned COG category. Within each group, C-terminal amino acid composition biases were computed at position −1, by comparing amino acid frequency to the frequency in the bulk of all sequences in the group. Significance of the biases was tested using Fisher's exact test and multiple testing correction with 5% false discovery rate. Categories A, B, W, and Z contained the lowest number of proteins (from 396 to 1,490, compared to 242,738 in the J category), which resulted in lower statistical power for the estimation of biases.

© 2020 The Authors

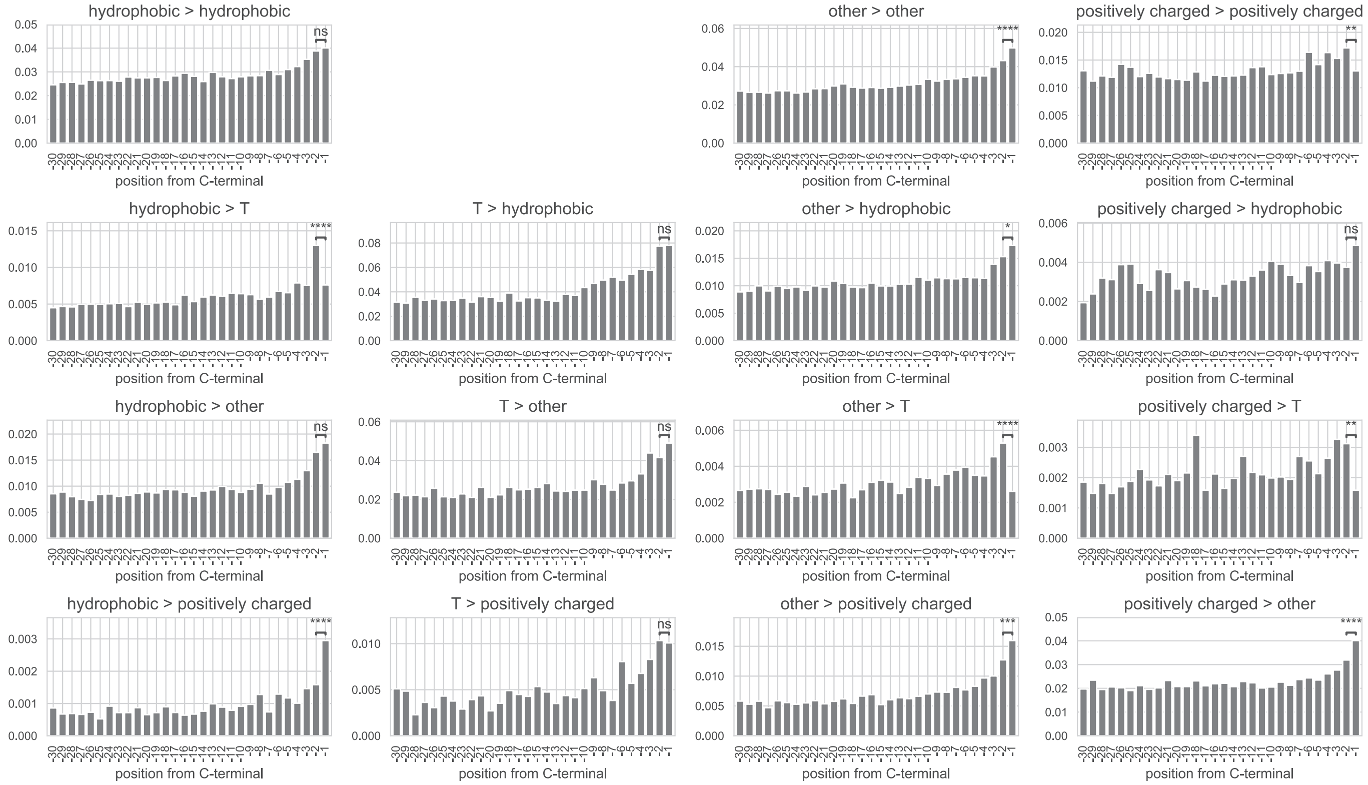

Figure EV3. Site-specific substitution rates between amino acid groups at the C-terminal.

Substitution rates between amino acid groups at each position from the C-terminal. In order to allow reliable statistics, amino acids were grouped into the following categories: positively charge (K, R), hydrophobic (A, I, L, M, F, W, Y, V), threonine (T), and others (H, D, E, N, Q, S, P, C, G). All 15 possible between-group and within-group substitutions are plotted. The same data were used as in Fig 4C. The difference of the substitution rate between positions −1 and −2 was tested by means of two-sided Fisher's test. Significance code: n.s. not significant for $P > 0.05$, * for $P < 0.05$, ** for $P < 0.01$, *** for $P < 0.001$, **** for $P <$ 1e-4.

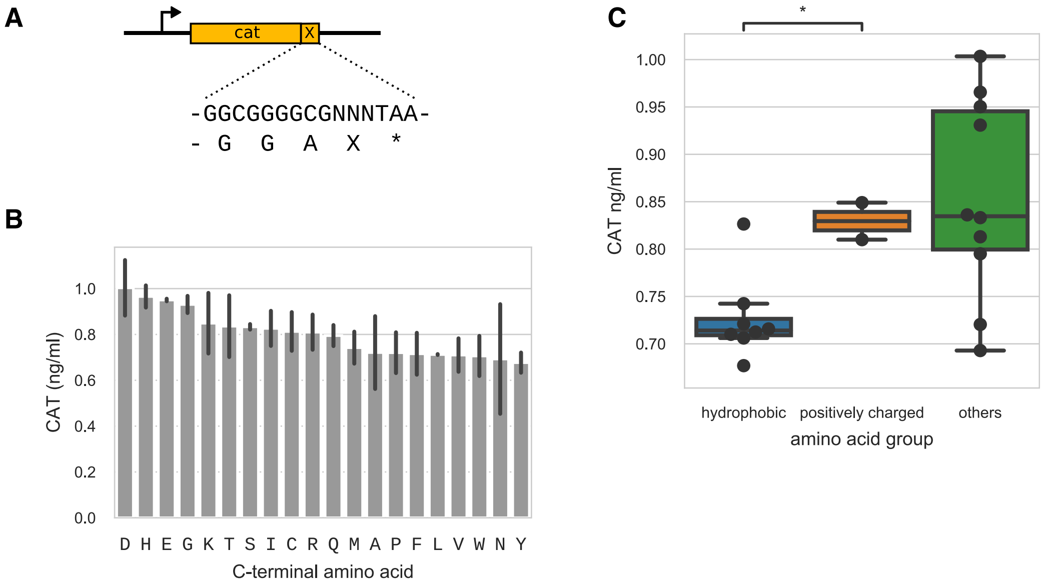

Figure EV4.

 © 2020 The Authors

◀ **Figure EV4. Protein quantification of the cat reporter gene with C-terminal amino acid composition variation.**

A One of the 20 amino acids was added to the C-terminal of the cat reporter gene and expressed in *Mycoplasma pneumoniae*.

B Protein quantification by ELISA antibody testing normalized by total protein abundance, for 2 biological replicates. Error bars show standard deviations.

C Protein expression levels grouped by amino acid properties. Difference between hydrophobic and positively charged amino acid groups was tested using independent *t*-test, *P = 0.015. Boxes show the quartiles, central lines show the median, and whiskers extend up to 1.5 times the interquartile range from either the high or low quartile. All 20 data points are also plotted.

© 2020 The Authors
